# Autoinflammatory disease and severe neutropenia due to *de novo* variant of PSTPIP1 with increased binding to pyrin

**DOI:** 10.70962/jhi.20250201

**Published:** 2026-01-23

**Authors:** Sarah Cook, Kranthi Nomula, Claire E. Cross, Hwi M. Gil, Joseph M. Choi, Saara Kaviany, James A. Connelly, Christopher C. Chang, Janet G. Markle

**Affiliations:** 1 Vanderbilt University School of Medicine, Nashville, TN, USA; 2 Division of Immunology Allergy and Rheumatology, Joe DiMaggio Children’s Hospital, Hollywood, FL, USA; 3 Department of Pathology, Microbiology and Immunology, Vanderbilt University Medical Center, Nashville, TN, USA; 4 Department of Pediatrics, Vanderbilt University Medical Center, Nashville, TN, USA; 5 Department of Medicine, Vanderbilt University Medical Center, Nashville, TN, USA; 6 Vanderbilt Institute for Infection, Immunology and Inflammation, Vanderbilt Center for Immunobiology, Vanderbilt Genetics Institute, Nashville, TN, USA

## Abstract

Mutations in the gene PSTPIP1 may cause several different autoinflammatory syndromes, but the mechanisms by which distinct PSTPIP1 mutations lead to these differing phenotypes are not fully understood. The two best characterized autoinflammatory conditions resulting from PSTPIP1 mutation are pyogenic arthritis, pyoderma gangrenosum, and acne (PAPA) syndrome and PSTPIP1-associated myeloid-related proteinemia inflammatory (PAMI) syndrome. Here, we report a novel gain-of-function PSTPIP1 mutation (p.N236K) causing PAMI syndrome in a patient with systemic autoinflammation and severe neutropenia. This mutant form of PSTPIP1 shows increased binding to pyrin and leads to heightened inflammasome formation, relative to WT PSTPIP1. We also identify a transcriptional signature in blood from PAMI patients suggestive of enhanced T cell activation and altered neutrophil survival and/or function. Further research on PSTPIP1-related autoinflammatory conditions is needed to more deeply understand the genetic and immunological drivers of disease and contribute to improving patient outcomes.

## Teaser

Cook et al. report a novel *de novo* heterozygous N236K mutation in PSTPIP1, identified in a patient with neonatal-onset PAMI that was ultimately fatal. The N236K mutation showed increased binding to pyrin and enhanced inflammasome formation. The authors also identify blood transcriptome changes in PAMI patients with both N236K and the more common E250K mutation.

While the spectrum of known proline-serine-threonine phosphatase-interacting protein 1 (PSTPIP1)–associated inflammatory diseases (PAIDs) has continued to expand over the last decade, the mechanistic basis of genotype–phenotype correlations remains unclear. The two best characterized PAIDs are pyogenic sterile arthritis, pyogenic gangrenosum, and acne (PAPA) syndrome and PSTPIP1-associated myeloid-related proteinemia inflammatory (PAMI) syndrome. PAPA syndrome is an autosomal dominant autoinflammatory disease that is characterized by sterile arthritis in early childhood and onset of dermatologic manifestations, including sterile ulcers and abscesses, around puberty (1). Compared to PAPA syndrome, patients with PAMI syndrome typically present earlier and with more severe disease. Clinical features include skin inflammation, arthritis, severe systemic inflammation, hepatosplenomegaly, failure to thrive, cytopenias, hyperzincemia, and high levels of the calprotectin subunits MRP8 and MRP14 (1). To date, only two heterozygous PSTPIP1 mutations, E250K and E257K, are known to cause PAMI. Here, we report a patient who presented with features of PAMI syndrome and was found to have a *de novo *variant leading to amino acid substitution N236K in PSTPIP1.

**Figure 1.**
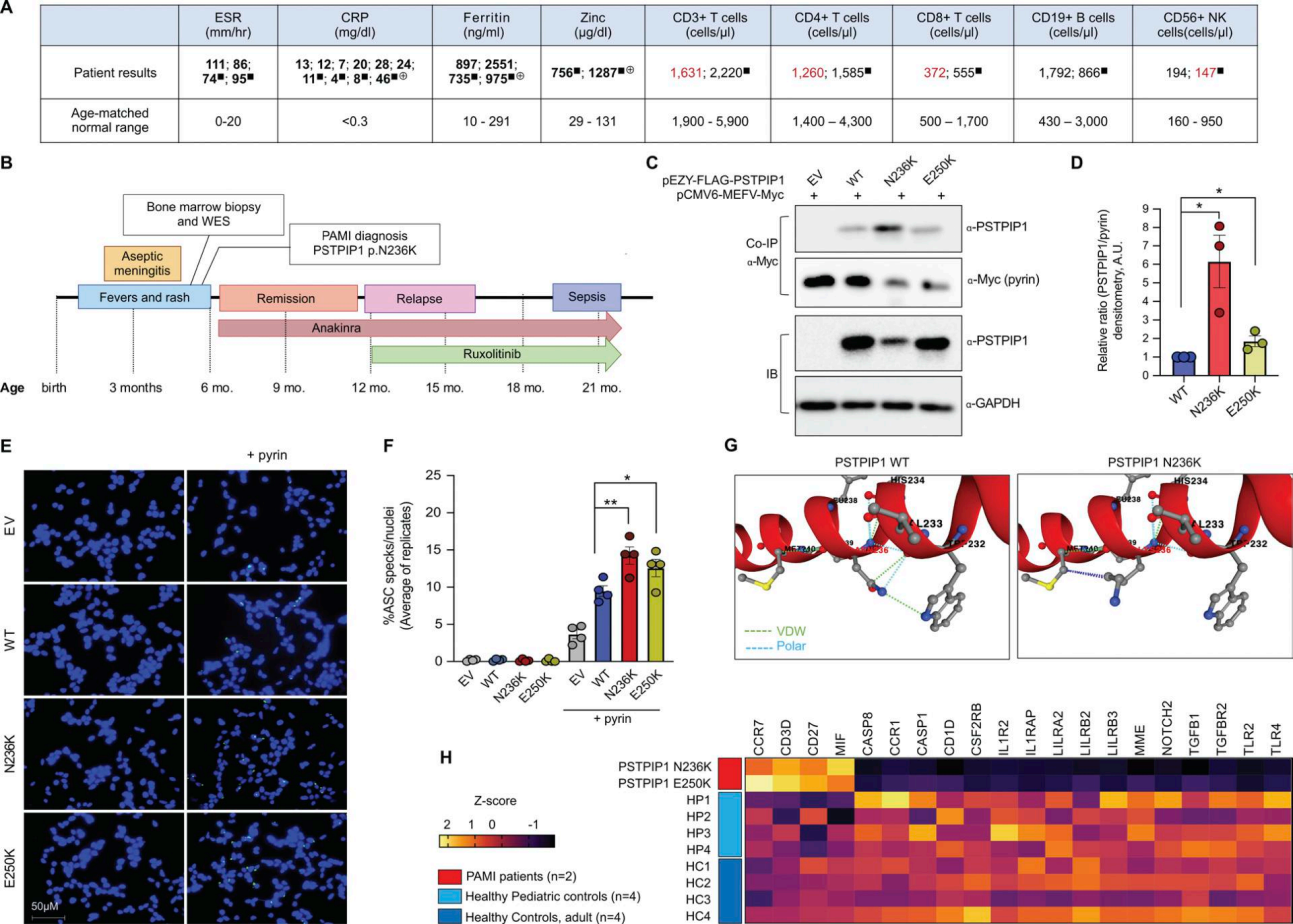
**Identification and functional characterization of PSTPIP1 mutation N236K in a patient with PAMI syndrome. (A)** Laboratory testing results for markers of inflammation and for leukocyte subset frequencies. Results were obtained on at least two occasions for erythrocyte sedimentation rate (ESR), C-reactive protein (CRP), ferritin, and zinc, and the indicated leukocyte subsets. Repeated measures obtained on separate dates are separated by semicolons. Values above the age-matched normal range are bolded. Values below the normal range are in red font. Data were obtained while the patient was between 5 and 22 mo of age. ^■^measurement taken while on anakinra; ^⊕^while on ruxolitinib. NK, natural killer. **(B)** Timeline of patient’s clinical course. WES, whole-exome sequencing. **(C)** Co-immunoprecipitation (co-IP) experiments assessing the ability of WT and mutant forms of PSTPIP1 (pEZY-FLAG-PSTPIP1) to bind pyrin (pCMV6-MEFV-Myc). EV, empty vector control; IB, immunoblot. **(D)** Quantitation of PSTPIP1 protein, relative to pyrin protein, in co-IP samples from three independent experiments performed as in B. *P < 0.05, paired two-tailed *T* test. **(E)** ASC speck assays using HEK293-ASC^YFP^ cells transfected with pEZY-FLAG-PSTPIP1 plasmids (EV, WT, N236K, or E250K), with or without pyrin (pCMV6-MEFV-Myc). ASC is visualized in green, and DAPI-stained nuclei are visualized in blue. **(F)** Quantitation of ASC specs, normalized to DAPI-stained nuclei, from fluorescence microscopy images using automated counting in ImageJ. Each data point is the average of at least 10 fields per condition in a single experiment. Graph shows results from *n* = 4 independent experiments. *P < 0.05, **P < 0.01, paired two-tailed *T* test. **(G)** A partial crystal structure of WT PSTPIP1 surrounding the N236 residue is shown, from Protein Data Bank entry 7AAN. The N236 amino acid is polar and undergoes Van der Waals interactions (VDW) with neighboring amino acids. Modeling of the N236K mutation shows loss of VDW interactions and polarity. **(H)** Patterns of gene expression in whole blood samples from PAMI patients (*n* = 2), healthy pediatric controls (HP, *n* = 4), and healthy adult controls (HC, *n* = 4). All genes included on heatmap showed P < 0.05 in comparisons of patients vs. healthy adult controls and patients vs. healthy pediatric controls, and P > 0.05 in comparisons of healthy adult controls vs. healthy pediatric controls (two-tailed unpaired *T* test). Source data are available for this figure: SourceData F1.

A female infant was born to healthy, non-consanguineous parents at 40 wk gestation after an uncomplicated pregnancy. The patient’s clinical course immediately after birth was unremarkable. Between the ages of 1–6 mo, the patient had multiple episodes of fever and generalized maculopapular rash. Laboratory findings revealed persistently elevated erythrocyte sedimentation rate, C-reactive protein, ferritin, and zinc (Fig. 1 A). She was also noted to have elevated soluble IL-2 receptor (3,513 U/ml, normal range 389–1,940), D-dimer (8.29 mg/L, normal range 0–0.49), CXCL9 (1,657 pg/ml, normal range <121) and IL-18 levels (50,285 pg/ml, normal range 89–540), and persistent neutropenia. Blood cell counts for major leukocyte subsets were slightly low on one occasion but normal on subsequent testing (Fig. 1 A). Antinuclear antibody testing was negative. There was concern for arthritis given significant patient discomfort when her lower extremities were manipulated for diaper care. The patient appropriately met developmental milestones, and neurologic evaluation was normal, including auditive testing. In the setting of her chronic neutropenia and recurrent fever, a bone marrow biopsy was performed and demonstrated areas with trilineage hematopoiesis with a left-shifted myeloid maturation and possible maturational arrest at the metamyelocyte level, small mature-appearing lymphocytes, and normoblastic erythroid precursors without atypia. Myeloid to erythroid ratio was within normal limits. No blast cells or overtly dysplastic hematopoietic cells were noted, nor was there evidence of hemophagocytic lymphohistiocytosis. The patient had no history of skin ulceration, acne, hepatosplenomegaly, or lymphadenopathy. The patient’s early age at presentation, severity of disease, and chronic neutropenia suggested a monogenic autoinflammatory syndrome (Fig. 1 B). Whole-exome sequencing revealed a heterozygous, *de novo* C > A variant at Chr15:77031245, which affects transcript NM_003978.5 (c.708C > A) and results in the missense variant p.N236K in PSTPIP1. This variant was initially classified as a variant of uncertain significance and was previously reported in a single patient with PAMI syndrome, but no functional studies were reported (2). Treatment with an IL-1 inhibitor anakinra (6 mg/day) initially controlled episodes of fever and rash, though fevers recurred after 5 months of treatment, despite dose escalation to 7.5 mg/day, then 10 mg/day over this period. Her neutropenia was treated with filgrastim with short-term success. At about a year of age, she was evaluated for bone marrow transplantation and began treatment with a Janus kinase (JAK) inhibitor ruxolitinib 2.5 mg twice daily as an initial dose, with an increase to 5 mg twice daily. Her symptoms generally improved while on anakinra and ruxolitinib, although her absolute neutrophil count on filgrastim ranged from 100 to 1,000/μl. At 22 mo of age, the patient presented in severe respiratory distress, likely secondary to metapneumovirus infection, and passed away rapidly despite maximum resuscitative efforts. Written informed consent was obtained for all participants, and samples for this study were collected under an approved protocol (Vanderbilt University Human Research Protections Program and Institutional Review Board).

Like the known PAMI-causal mutations E250K and E257K, the N236K variant is proximal to the coiled-coil domain of PSTPIP1, which binds pyrin (1). Pyrin recruits PSTPIP1 into protein complexes containing the apoptosis-associated speck-like protein (ASC), leading to inflammasome assembly and caspase-1–dependent IL-18 and IL-1β secretion (1). To evaluate the impact of N236K on pyrin inflammasome activity, WT or mutant forms of PSTPIP1 (pEZY-FLAG-PSTPIP1) were ectopically co-expressed with pyrin (pCMV6-MEFV-Myc) in HEK293T cells. Myc-tagged pyrin was used for co-immunoprecipitation, and western blotting was performed for PSTPIP1. Mutant N236K PSTPIP1 showed enhanced pyrin binding relative to WT PSTPIP1, which surpassed that of the E250K mutation (Fig. 1 C). Quantitation of normalized PSTPIP1/pyrin signals by densitometry (ImageJ) is shown for three independent replicates (Fig. 1 D). To assess pyrin inflammasome activation, WT or mutant forms of PSTPIP1 were expressed, with or without pyrin (pCMV6-MEFV-Myc) in HEK293-ASC^YFP^ cells stably expressing a yellow fluorescent protein (YFP)–tagged ASC protein, which oligomerizes to form “specks” upon inflammasome activation. ASC^YFP^ specks and DAPI-stained nuclei were visualized on an Olympus BX51 microscope with a 40× objective, and a minimum of six fields were captured per condition. Automated counting of ASC^YFP^ specks and nuclei (ImageJ) was performed on >100 nuclei per condition in each experiment and results plotted for four replicate experiments. In the absence of pyrin, neither WT nor PAMI-associated versions of PSTPIP1 induced inflammasome activation (Fig. 1, E and F). When co-expressed with pyrin, the N236K and E250K mutant forms of PSTPIP1 induced increased pyrin inflammasome activation relative to WT (Fig. 1, E and F). Collectively, these data suggest that the N236K variant alters PSTPIP1 protein structure, allowing for increased pyrin binding and pyrin inflammasome nucleation. Accordingly, modelling of the structural impact of the N236K variant using PremPS predicted a change in unfolding free energy of 0.74 kcal/mol, indicating a destabilizing variant, and loss of non-covalent interactions with nearby amino acids (Fig. 1 G).

To investigate the mechanisms of immune dysregulation resulting from the N236K variant, we profiled transcriptional changes in whole blood samples from this patient, another PAMI patient with the published E250K variant, healthy pediatric controls (*n* = 4), and healthy adult controls (*n* = 4). Blood samples from PAMI patients were collected during periods free of fever or rashes. Blood was collected in PAXgene Blood RNA tubes (#762165; BD), and RNA was extracted (#K0732; Thermo Fisher Scientific). Gene expression was measured using an nCounter Immunology Panel (#XT-CSO-HIM2-12; NanoString). Raw count files were normalized (nSolver Software v4) using the geometric mean of 15 housekeeping genes, per the manufacturer’s protocol. Normalized expression data were exported for further analysis. Two-tailed unpaired *T* tests were performed to compare gene expression between two groups, and Z-scores were calculated and used for heatmap (GraphPad Prism). PAMI patients’ samples showed elevated expression of several genes involved in T cell receptor (TCR) activation or co-stimulation (*CCR7*, *CD3D*, and *CD27*) and reduced expression of genes important for neutrophil survival, activation, and function (*CSF2RB*, *CCR1*, *LILRA2*, *LILRB2*, and *LILRB3*) and T regulatory cell function (*TGFB1* and *TGFBR2*) (Fig. 1 H). Prior work has shown that PSTPIP1 is recruited to the TCR complex and regulates F-actin remodeling following T cell activation (3), but it is not yet known whether this process is perturbed in PAMI. Additional studies are required to better understand how various PSTPIP1-dependent pathways and different cell types culminate in PAMI syndrome pathogenesis.

The N236K variant adds to the growing body of evidence that diverse mutations in *PSTPIP1* underlie a spectrum of autoinflammatory phenotypes. This study is limited by lack of data regarding MRP8 and MRP14 levels in this patient, as well as unavailability of viable primary cells to directly test for myeloid production of pro-inflammatory cytokines upon stimulation. Despite an increasing awareness and diagnosis of PAIDs, including PAMI, our mechanistic understanding of pathogenesis is limited, and the clinical heterogeneity of PAIDs may complicate reaching a diagnosis and delay proper treatment. Mouse strains with *Pstpip1* knocked out, or specific variants knocked in, do not recapitulate some hallmark phenotypes of PAID, limiting the utility of these mice for modelling PAID pathogenesis (4). In addition to affecting pyrin inflammasome activation, several PAID-causing variants disrupt the interaction of PSTPIP1 with the protein tyrosine phosphatase LYP (encoded by *PTPN22*) (5), a negative regulator of TCR activation; thus, potential roles for PSTPIP1–LYP interactions in regulating T cell function in PAMI merit further investigation. The severity of PAMI syndrome and the risk of early morbidity necessitate a high index of suspicion and clinician awareness of monogenic autoinflammatory phenotypes and genetic testing. The mainstay of treatment for PAMI syndrome and other related PAIDs include steroids, tumor necrosis factor inhibitors, and IL-1 inhibitors. More recently, JAK inhibitors were reported to control disease activity in patients with classical PAPA syndrome (4). While continued reporting of new cases and associated PAID mutations is necessary to fully understand these complex diseases, there is also a pressing need to explore the molecular mechanisms of disease and potential new therapeutic targets.

## Supplementary Material

SourceData F1 is the source file for Fig. 1.

## Data Availability

The data underlying Fig. 1 are available in the published article and from the corresponding author on request.
