# Supplementary figures and images for: Autoinflammatory disease and severe neutropenia due to de novo variant of PSTPIP1 with increased binding to pyrin
Source: J Hum Immun. 2026 Jan 23;2(2):e20250201. doi: 10.70962/jhi.20250201 (PMC13088949; doi:10.70962/jhi.20250201)

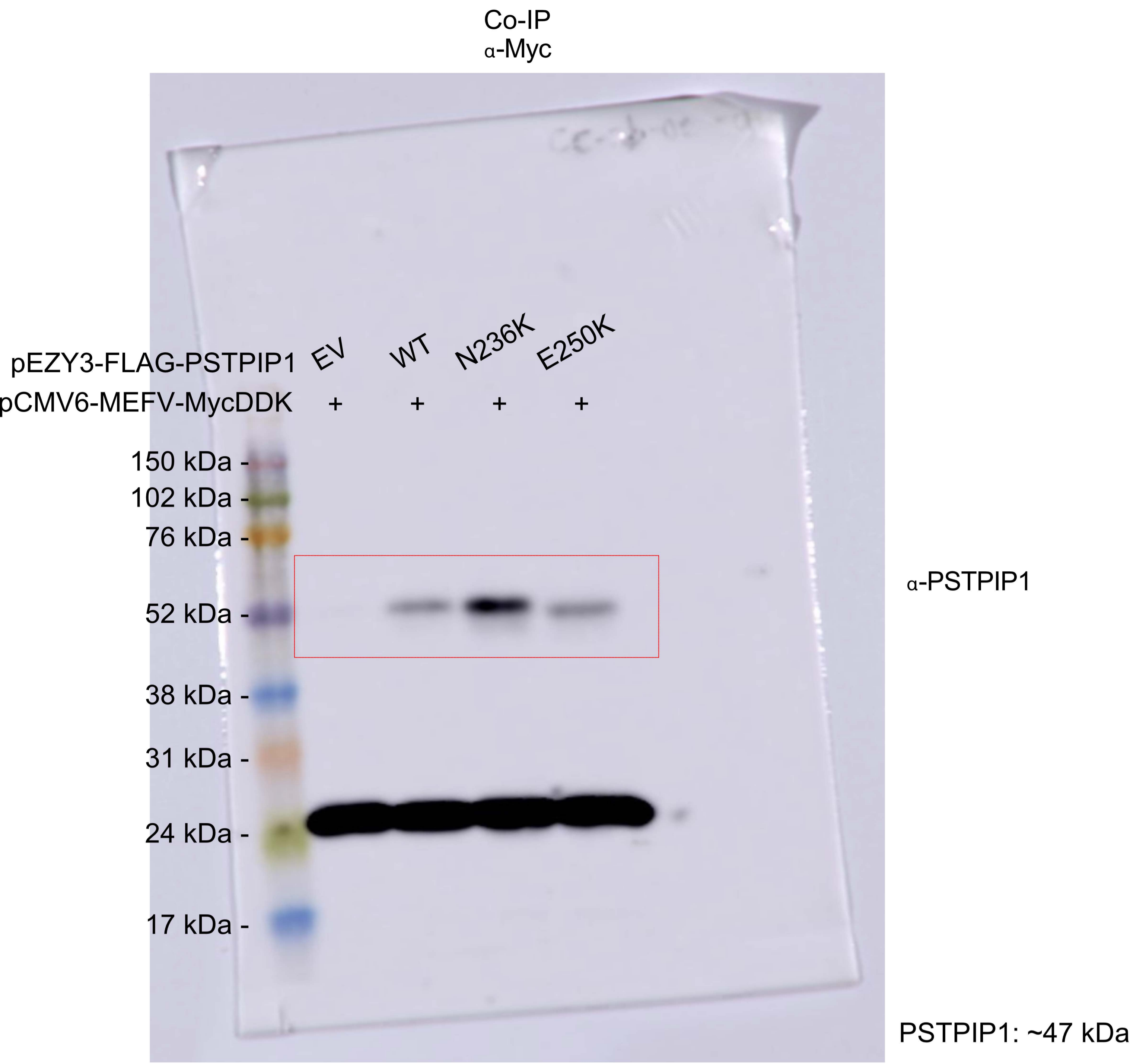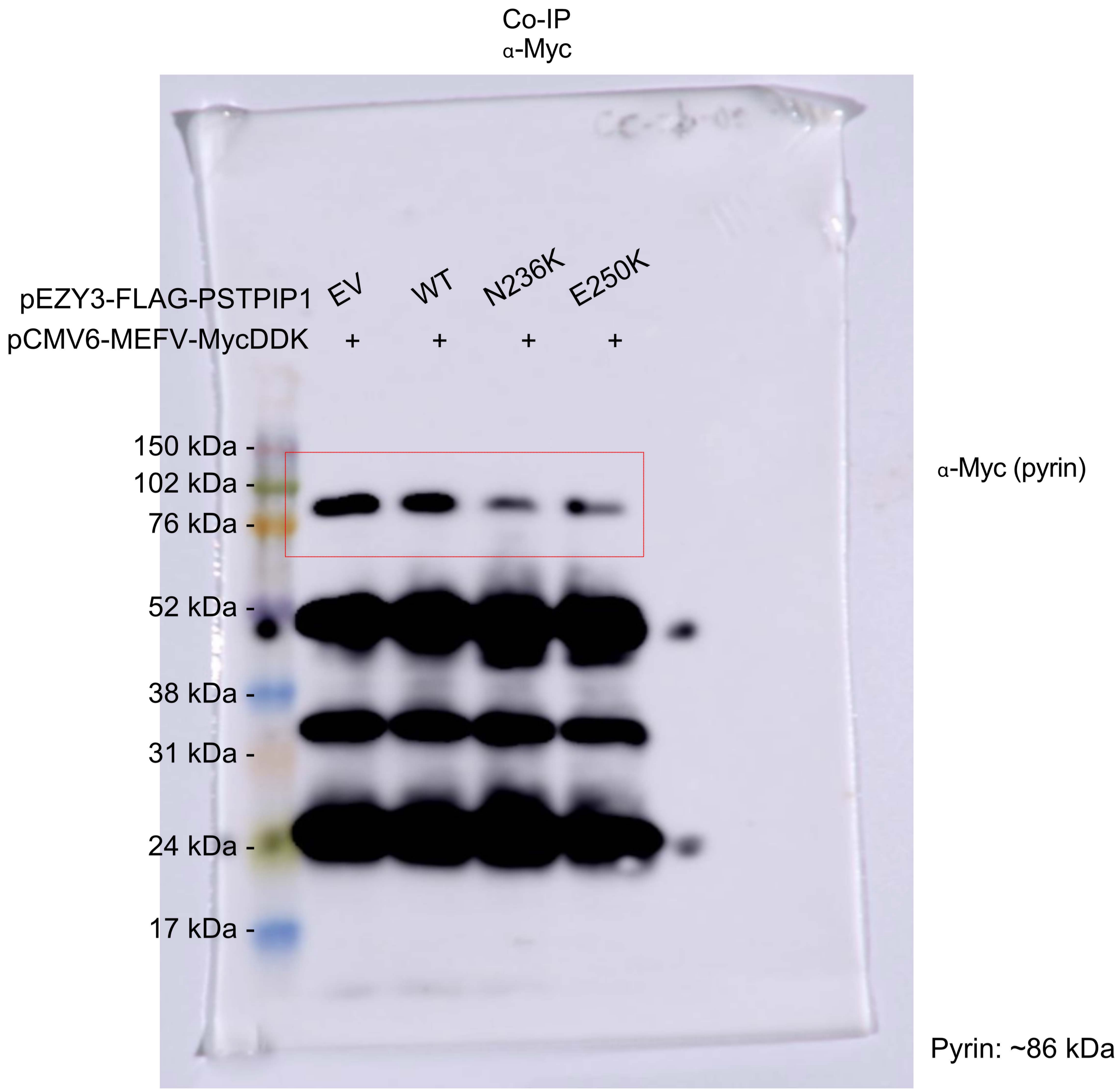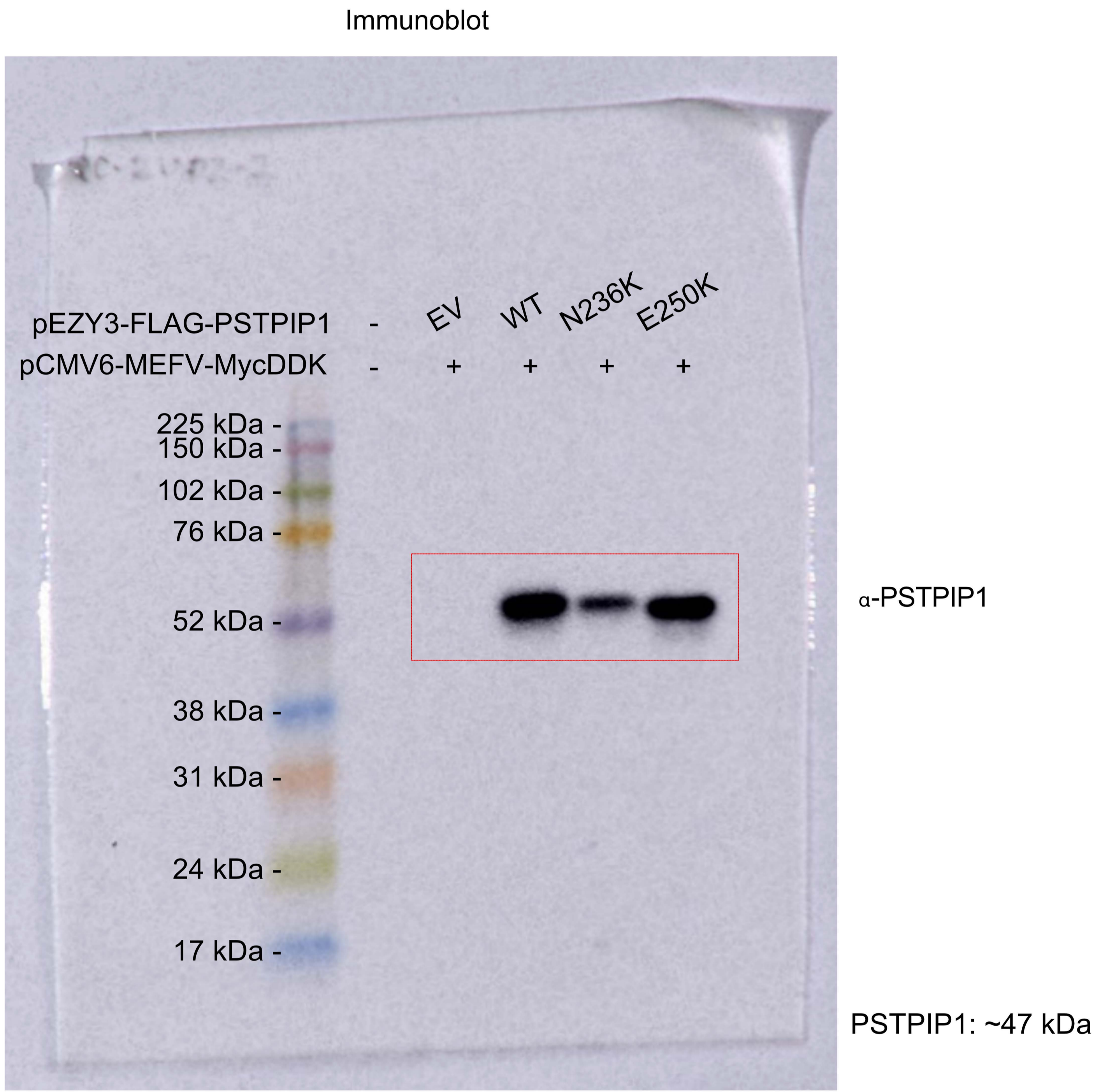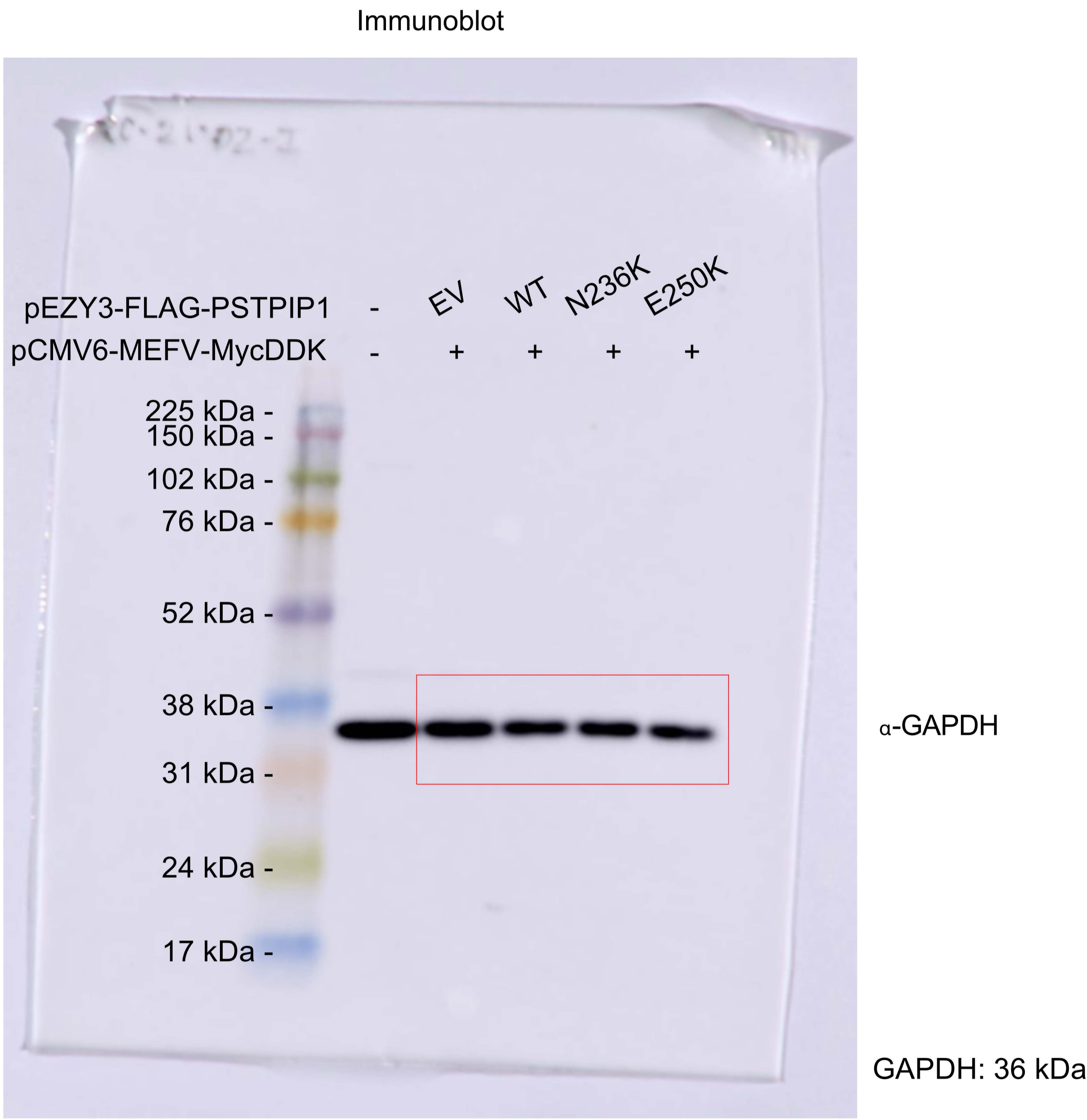

Supplement: SourceData F1 — is the source file for Fig. 1. [file jhi_20250201_sourcedataf1.pdf]
